# Bioinformatics analysis of photoexcited natural flavonoid glycosides as the inhibitors for oropharyngeal HPV oncoproteins

**DOI:** 10.1186/s13568-024-01684-6

**Published:** 2024-03-11

**Authors:** Maryam Pourhajibagher, Abbas Bahador

**Affiliations:** 1 Dental Research Center, Dentistry Research Institute, Tehran University of Medical Sciences, Tehran, Iran; 2 Department of Microbiology, School of Medicine, Tehran University of Medical Sciences, Tehran, Iran; 3 Fellowship in Clinical Laboratory Sciences, BioHealth Lab, Tehran, Iran

**Keywords:** HPV, Molecular docking, Virtual screening, ADMET properties, *In silico*

## Abstract

The presence of oropharyngeal human papillomavirus (HPV)-18 E6 and E7 oncoproteins is highly significant in the progression of oropharyngeal cancer. Natural flavonoid compounds have potential as photosensitizers for light-activated antimicrobial therapy against HPV-associated oropharyngeal cancer. This study evaluated five natural flavonoid glycosides including Fisetin, Kaempferol, Morin, Myricetin, and Quercetin as photosensitizers against HPV-18 E6 and E7 oncoproteins using computational methods. After obtaining the amino acid sequences of HPV-18 E6 and E7, various tools were used to predict and verify their properties. The PubChem database was then examined to identify potential natural flavonoid glycosides, followed by predictions of their drug-likeness and ADMET properties. Subsequently, molecular docking was conducted to enhance the screening accuracy and to gain insights into the interactions between the natural compounds and the active sites of HPV-18 E6 and E7 oncoproteins. The protein structures of E6 and E7 were predicted and validated to be reliable. The results of molecular docking demonstrated that Kaempferol exhibited the highest binding affinity to both E6 and E7. All compounds satisfied Lipinski's rules of drug-likeness, except Myricetin. They showed high absorption, distribution volume and similar ADMET profiles with no toxicity. In summary, natural flavonoid glycosides, especially Kaempferol, show potential as photosensitizers for antimicrobial photodynamic therapy against HPV-associated oropharyngeal cancer through inhibition of E6 and E7 oncoproteins. These findings provide insights into the development of novel therapeutic strategies based on antimicrobial photodynamic therapy.

## Introduction

Oropharyngeal human papillomavirus-18 (HPV-18), a specific type of HPV, has the potential to induce cancer in the oropharynx, which encompasses the tonsils and the base of the tongue. (Poelman et al. 2021). The HPV-18 virus produces two oncoproteins, E6 and E7, that play a crucial role in the development of cancer (Dong et al. 2020). The HPV E6 oncoprotein is recognized for its crucial involvement in the advancement of oropharyngeal cancer associated with HPV (Tomaić et al. 2016). The capacity of the virus to bind to and degrade the p53 tumor suppressor protein, along with its role in promoting cell proliferation and suppressing apoptosis, plays a significant role in the progression of cancer by fostering genetic mutations and destabilizing the genome (Tomaić et al. 2016; Vats et al. 2021; Li et al. 2019). Therefore, targeting the E6 oncoprotein can be an attractive strategy for the development of novel therapeutic approaches against HPV-associated oropharyngeal cancer. Similarly, the E7 oncoprotein targets and degrades another tumor suppressor protein, called retinoblastoma protein (pRb). This also allows cells to divide and proliferate uncontrollably, leading to the development of cancer (Gonzalez et al. 2001; Pešut et al. 2021).

The standard treatment of oropharyngeal HPV has been concurrent radiation and chemotherapy, but upfront surgical approaches are becoming more common due to advances in transoral surgery techniques (Forastiere 2008). Despite the high survival rates, patients frequently encounter enduring toxicity and unfavorable functional outcomes due to the critical role of the oropharynx in daily activities (Gillison et al. 2012; Ward et al. 2016). Both surgical and medical interventions can lead to notable morbidity, often resulting in persistent dysphagia and speech difficulties as common consequences. Additional side effects encompass nausea, vomiting, dry mouth, dental complications, loss of taste, and challenges in mouth opening, among various others (Nutting et al. 2011; Irune et al. 2014; Kocak-Uzel et al. 2014; Timbang et al. 2019). Antimicrobial photodynamic therapy (aPDT) is a promising emerging approach for treating diverse infectious diseases, including those attributed to HPV (Rosa and Silva 2014; Songca 2023). aPDT is a therapeutic technique that employs a photosensitizing agent called photosensitizer, light, and oxygen to produce reactive oxygen species (ROS) capable of eliminating microbial cells. The photosensitizer is absorbed by the microbial cells and activated when exposed to specific light wavelengths, triggering the generation of ROS inside the cells. These ROS can inflict damage upon microbial cell membranes, proteins, and DNA, ultimately leading to the demise of the cells (Pourhajibagher and Bahador 2019; Rees et al. 2023).

aPDT has the potential to effectively combat both enveloped and non-enveloped viruses. Enveloped viruses possess a viral particle enclosed by a lipid membrane, which can be damaged by ROS generated during aPDT, leading to the inactivation of the virus. Non-enveloped viruses, on the other hand, do not have a lipid membrane but have a protein shell that surrounds the viral nucleic acid. During aPDT, the protein shell of non-enveloped viruses can be damaged by ROS, which can disrupt the structure of the virus and prevent it from infecting host cells (Almeida et al. 2020; Fekrazad et al. 2021).

The considerable anticancer and antiviral properties exhibited by natural flavonoid glycosides (Kopustinskiene et al. 2020; Badshah et al. 2021) make them promising candidates for the creation of innovative agents for aPDT. Natural flavonoid glycosides are a group of compounds found in various plants. Myricetin, morin, fisetin, Kaempferol, and Quercetin are five examples of natural flavonoid glycosides that have been extensively studied and are known to exhibit potent biological activities (Panche et al. 2016).

In this paper, we present a comprehensive bioinformatics analysis of natural flavonoid glycosides-mediated aPDT as inhibitors for oropharyngeal HPV-18 E6 and E7 oncoproteins. The study employs a range of computational tools to predict the binding affinity, mode of binding, and stability of the interaction between selected flavonoid glycosides and E6 and E7 oncoproteins. To the best of our understanding, this research represents the initial exploration of the capability of natural flavonoid glycosides-mediated aPDT as inhibitors of oropharyngeal HPV-18 E6 and E7 oncoproteins through bioinformatics methodologies. The outcomes of this study hold the potential to offer valuable insights into the advancement of efficacious therapeutic strategies for HPV-related oropharyngeal cancer, centered around aPDT.

## Materials and methods

### Retrieval of amino acid sequences

The complete amino acid sequences of HPV-18 E6 and E7 oncoproteins, having the accession numbers AAK95792 and AAK95798, respectively, were obtained from the National Center for Biotechnology Information (NCBI) database (https:// www.ncbi.nlm.nih.gov/). The Protein-Basic Local Alignment Search Tool (BLASTP; http://blast.ncbi.nlm.nih.gov/Blast) was utilized to analyze the sequences of HPV-18 E6 and E7 oncoproteins in order to identify an appropriate template.

### Prediction of physicochemical parameters

The physicochemical characteristics of HPV-18 E6 and E7 oncoproteins were obtained using the ProtParam tool (https://web.expasy.org/protparam/). Additionally, the ProtScale tool (http://web.expasy.org/protscale/) was utilized to calculate the average hydrophobicity and other physical and chemical properties, providing insights into the hydrophilicity and hydrophobicity of the proteins.

### Prediction of the secondary and tertiary structures

The SOPMA server (https://npsa-prabi.ibcp.fr/cgi-bin/npsa_automat.pl?page=/NPSA/npsa_sopma.html), which employs the Self-Optimized Prediction Method with Alignment, was utilized to predict the secondary structure of HPV-18 E6 and E7 oncoproteins. Furthermore, the three-dimensional structure of HPV-18 E6 and E7 oncoproteins, with PDB IDs 4GIZ and 6IWD respectively, was obtained from the Molecular Modeling Database (MMDB; https://www.ncbi.nlm.nih.gov/Structure/MMDB/mmdb.shtml). Additionally, the interactions of the ligands and metals in each oncoprotein with their surrounding amino acids were investigated through PDBsum (http://www.ebi.ac.uk/pdbsum).

### Model quality assessment

The reliability of the generated structures was assessed by analyzing their Ramachandran scores with the PROCHECK server (https://www.ebi.ac.uk/thornton-srv/software/PROCHECK/), ERRAT (https://saves.mbi.ucla.edu/), and ProSA-web (https://prosa.services.came.sbg.ac.at/prosa.php) servers. Additionally, the SWISS-MODEL Structure Assessment tool (https://swissmodel.expasy.org/assess) and QMEAN tool (https://swissmodel.expasy.org/qmean/) were employed together to estimate the QMEAN Z-score and overall quality of the model. Moreover, signal peptides cleavage sites in HPV-18 E6 and E7 oncoproteins were identified using SignalP—5.0 server (https://services.healthtech.dtu.dk/services/SignalP-5.0/).

### Molecular dynamics simulation

To evaluate the stability of the oncoproteins HPV-18 E6 and E7, a molecular dynamics simulation was conducted using the iMOD server (https://imods.iqfr.csic.es/). The stability of the proteins was analyzed using different methods, such as the main-chain deformability plot, B-factor, eigenvalue, covariance map, and elastic network model.

### Prediction of functional classification

To classify the functional aspects of the HPV-18 E6 and E7 oncoproteins, their biological process (BP), molecular function (MF), and cellular component (CC) were examined. The gene ontology (GO) terms were utilized as the selected system for functional classification (https://zhanglab.dcmb.med.umich.edu/C-I-TASSER/).

### Collection of ligands

For this study, five natural photosensitizers were chosen as ligands. These photosensitizers are: Fisetin (2-(3,4-dihydroxyphenyl)-3,7-dihydroxychromen-4-one), Kaempferol (3,5,7-trihydroxy-2-(4-hydroxyphenyl)chromen-4-one), Morin (2-(2,4-dihydroxyphenyl)-3,5,7-trihydroxychromen-4-one), Myricetin (3,5,7-trihydroxy-2-(3,4,5-trihydroxyphenyl)chromen-4-one), and Quercetin (2-(3,4-dihydroxyphenyl)-3,5,7-trihydroxychromen-4-one). The SDF structures of these compounds were obtained from the DrugBank (https://go.drugbank.com/) and PubChem (http://pubchem.ncbi.nlm.nih.gov) databases.

### Molecular docking

To conduct molecular docking analysis, the elimination of water molecules and other heteroatoms like phosphate molecules was carried out. The HPV-18 E6 and E7 oncoproteins were then subjected to docking with Fisetin, Kaempferol, Morin, Myricetin, and Quercetin using the CB-Dock2 server (https://cadd.labshare.cn/cb-dock2). Multiple conformers of the ligands were generated during the docking process, and the docking complexes with the most favorable free binding energy conformations (with the lowest kcal/mol) were considered as the best interactions between the target receptors and ligands.

### Drug-likeness profile

In order to evaluate the drug-like properties of natural flavonoid glycosides, the Molinspiration server (https://www.molinspiration.com/cgi-bin/properties) was utilized for analysis. To be considered "drug-like," the candidate ligands needed to meet specific criteria, including adherence to Lipinski's rule of five. This rule emphasizes the importance of maintaining a balance between hydrophilicity and lipophilicity. The criteria for Lipinski's rule of five include having fewer than five hydrogen bond donors, fewer than ten hydrogen bond acceptors, a molecular weight below 500 g/mol, and an octanol/water partition coefficient (LogP) below 5. Additionally, a drug with a topological polar surface area (TPSA) value below 140 Å2 has the potential to be absorbed over 90%. The number of rotatable bonds also plays a role in a molecule's absorptive capabilities.

### *In silico* pharmacokinetics/pharmacodynamics evaluation

The ADMET profile of natural flavonoid glycosides was assessed using the SwissADME tool (http://www.swissadme.ch/) and the pkCSM-pharmacokinetics tool (http://structure.bioc.cam.ac.uk/pkcsm) *in silico*.

## Results

### Retrieval of HPV-18 E6 and E7 oncoproteins

Using BLASTP to search for similarities with target proteins found in HPV-18, the findings revealed that the E6 and E7 oncoproteins share similarities with the protein structures represented by PDB IDs 4GIZ and 6IWD, respectively. The protein alignment between E6 and 4GIZ resulted in a total score of 50.4 with 87% query cover and 24.09% identity. Similarly, the protein alignment between E7 and 6IWD showed a query cover, identity, and total score of 37%, 38.46%, and 35, respectively.

### Physicochemical properties of HPV-18 E6 and E7 oncoproteins

**Fig. 1:**
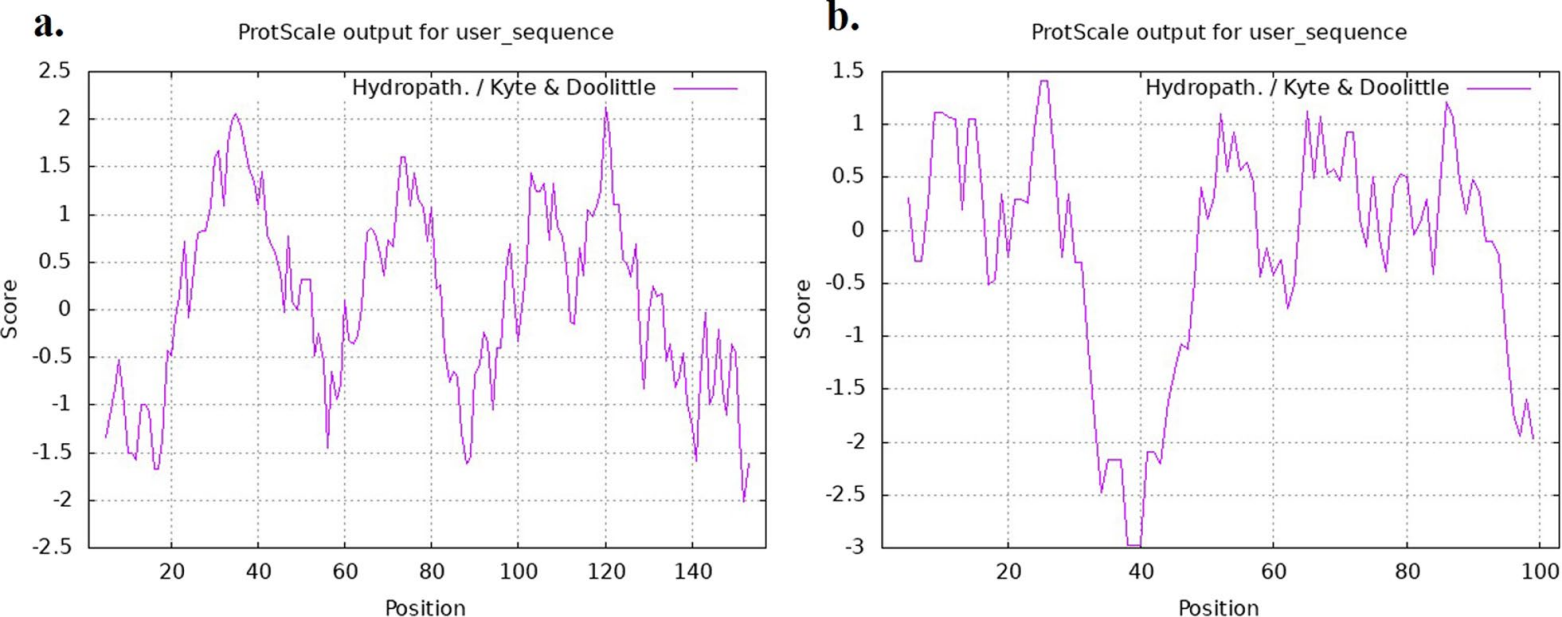
Kyte and Doolittle hydropathy plot: **a** HPV-18 E6 oncoprotein, **b** HPV-18 E7 oncoprotein. A positive peak indicates a probability that the corresponding polypeptide fragment is hydrophobic

The HPV-18 E6 oncoprotein is characterized by its length of 157 amino acids and molecular mass of 17920.75 Da. It has a theoretical isoelectric point (pI) value of 5.37 and consists of 16 strong basic (+) amino acids (K, R) and 20 strong acidic (−) amino acids (D, E). Its atomic composition is C_804_H_1228_N_208_O_227_S_15_, and it exhibits an instability index of 33.41, indicating its stability as a protein. The average hydrophilicity coefficient GRAVY is 0.053, suggesting it is a hydrophobic protein. The hydrophilic nature is further supported by the analysis of the Kyte and Doolittle hydropathy plot (Fig. 1a). On the other hand, the HPV-18 E7 oncoprotein consists of 103 amino acids with a molecular mass of 11699.37 Da. It has a theoretical pI value of 4.47 and contains 9 strong basic (+) amino acids (K, R) and 21 strong acidic (−) amino acids (D, E). The atomic composition is C_510_H_814_N_136_O_162_S_8_, and it exhibits an instability index of 52.66, classifying it as an unstable protein. The average hydrophilicity coefficient GRAVY is − 0.197, indicating it is a hydrophilic protein. This hydrophilic nature is also supported by the Kyte and Doolittle hydropathy plot (Fig. 1b). A comprehensive physiochemical profile of these proteins is presented in Table 1.

**Table 1:** Molecular and physiochemical properties of HPV-18 E6 and E7 oncoproteins

Physiochemical profiling	E6		E7	
Molecular weight	17920.75		11699.37	
Theoretical pI	5.37		4.47	
Total number of negatively charged residues (Asp + Glu)	20		21	
Total number of positively charged residues (Arg + Lys)	16		9	
Atomic composition	Carbon	804	Carbon	510
	Hydrogen	1228	Hydrogen	814
	Nitrogen	208	Nitrogen	136
	Oxygen	227	Oxygen	162
	Sulfur	15	Sulfur	8
Formula	C_804_H_1228_N_208_O_227_S_15_		C_510_H_814_N_136_O_162_S_8_	
Total number of atoms	2482		1630	
Extinction coefficients in H_2_O (280 nm)	27,805		1865	
Estimated half-life: The N-terminal of the sequence considered is M (Met)	30 h (mammalian reticulocytes, in vitro) > 20 h (yeast, in vivo) > 10 h (*Escherichia coli*, in vivo)		30 h (mammalian reticulocytes, in vitro) > 20 h (yeast, in vivo) > 10 h (*Escherichia coli*, in vivo)	
Instability index (II)	33.41		52.66	
Aliphatic index	88.28		97.38	
Grand average of hydropathicity (GRAVY)	0.053		− 0.197	

### Prediction of secondary structures

To determine the secondary structures of the HPV-18 E6 and E7 oncoproteins, SOPMA was employed. The analysis unveiled that the HPV-18 E6 oncoprotein consisted of an alpha helix content of 57.32%, a random coil content of 26.11%, an extended strand content of 9.55%, and a beta turn content of 7.1%. In contrast, the HPV-18 E7 oncoprotein exhibited a random coil content of 49.51%, an alpha helix content of 32.04%, an extended strand content of 16.50%, and a beta turn content of 1.94%.

### Prediction of tertiary structures

**Fig. 2:**
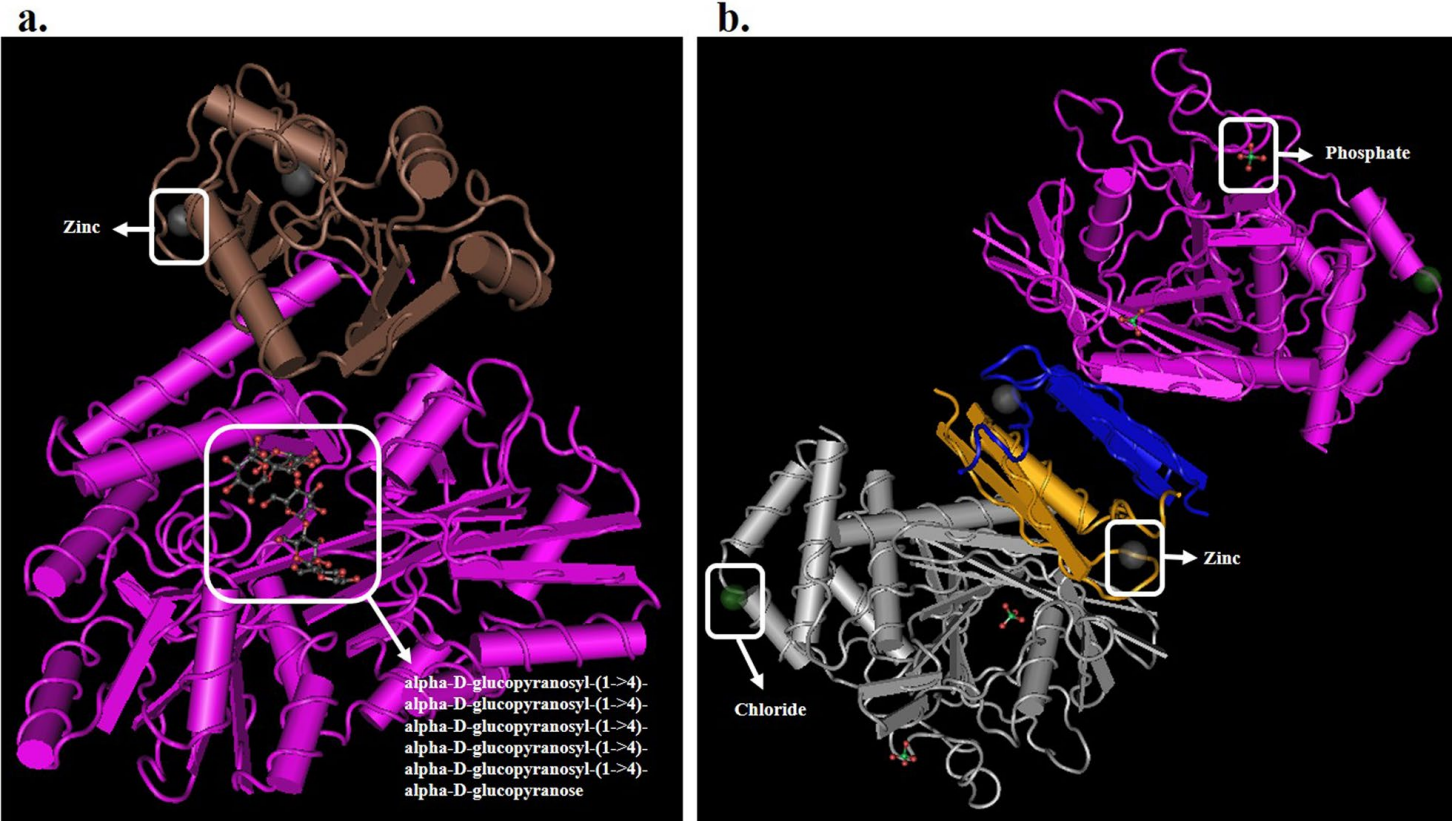
Three-dimensional structure of proteins: **a** HPV-18 E6 oncoprotein (PDB ID: 4GIZ), **b** HPV-18 E7 oncoprotein (PDB ID: 6IWD). The HPV-18 E6 protein consists of a complex structure, including multiple alpha-D-glucopyranosyl chains and two zinc ions, and the HPV-18 E7 protein is composed of four chains, four phosphate ions, two chloride ions, and two zinc ions

Using the MMDB program, the tertiary structures of the HPV-18 E6 and E7 oncoproteins were obtained and are illustrated in Fig. 2. The HPV-18 E6 oncoprotein, with a resolution of 2.55 Å, is composed of two chains, one molecule of alpha-D-glucopyranosyl-(1- > 4)-alpha-D-glucopyranosyl-(1- > 4)-alpha-D-glucopyranosyl-(1- > 4)-alpha-D-glucopyranosyl-(1- > 4)-alpha-D-glucopyranosyl-(1- > 4)-alpha-D-glucopyranose, and two zinc ions. Meanwhile, the HPV-18 E7 oncoprotein, with a resolution of 1.80 Å, has four chains, four phosphate ions, two chloride ions, and two zinc ions.

**Fig. 3:**
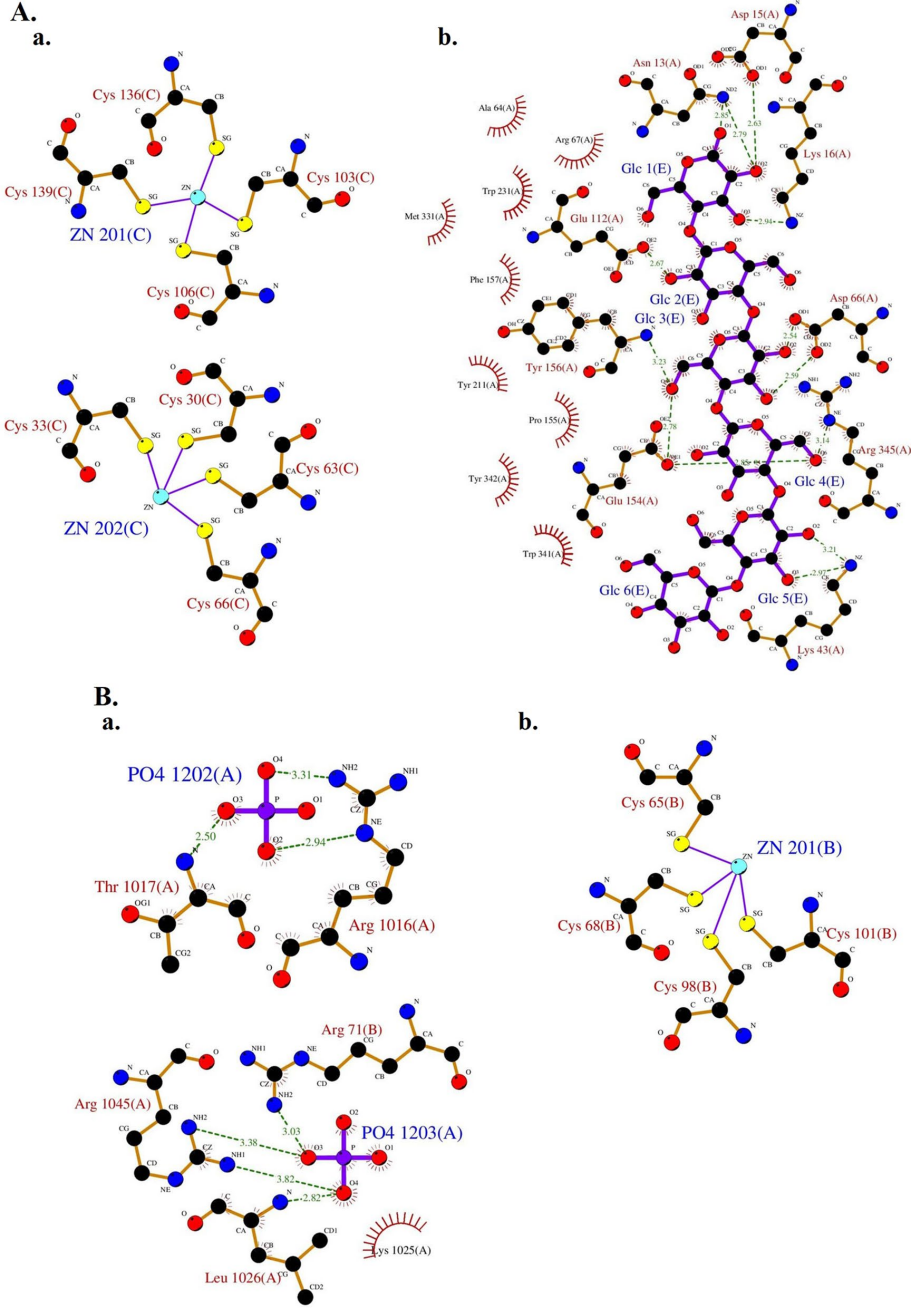
Interactions of the ligands and/or metals in: **A** HPV-18 E6 oncoprotein with their surrounding amino acids; **a** zinc ions (Zn), **b** alpha-D-glucopyranosyl-(1- > 4)-alpha-D-glucopyranosyl-(1- > 4)-alpha-D-glucopyranosyl-(1- > 4)-alpha-D-glucopyranosyl-(1- > 4)-alpha-D-glucopyranosyl-(1- > 4)-alpha-D-glucopyranose. **B** HPV-18 E7 oncoprotein with their surrounding amino acids; a. phosphate ions (PO_4_), b. zinc ion (Zn)

Figure 3 illustrates the connections between zinc ions (Image a in Fig. 3A) and alpha-D-glucopyranosyl-(1- > 4)-alpha-D-glucopyranosyl-(1- > 4)-alpha-D-glucopyranosyl-(1- > 4)-alpha-D-glucopyranosyl-(1- > 4)-alpha-D-glucopyranosyl-(1- > 4)-alpha-D-glucopyranose (Image b in Fig. 3A) in HPV-18 E6 oncoprotein, along with their neighboring amino acids, as well as the associations of phosphate ions (Image a in Fig. 3B) and zinc ion (Image b in Fig. 3B) in HPV-18 E7 with their surrounding amino acids.

### Validation of predicted tertiary structures

**Fig. 4:**
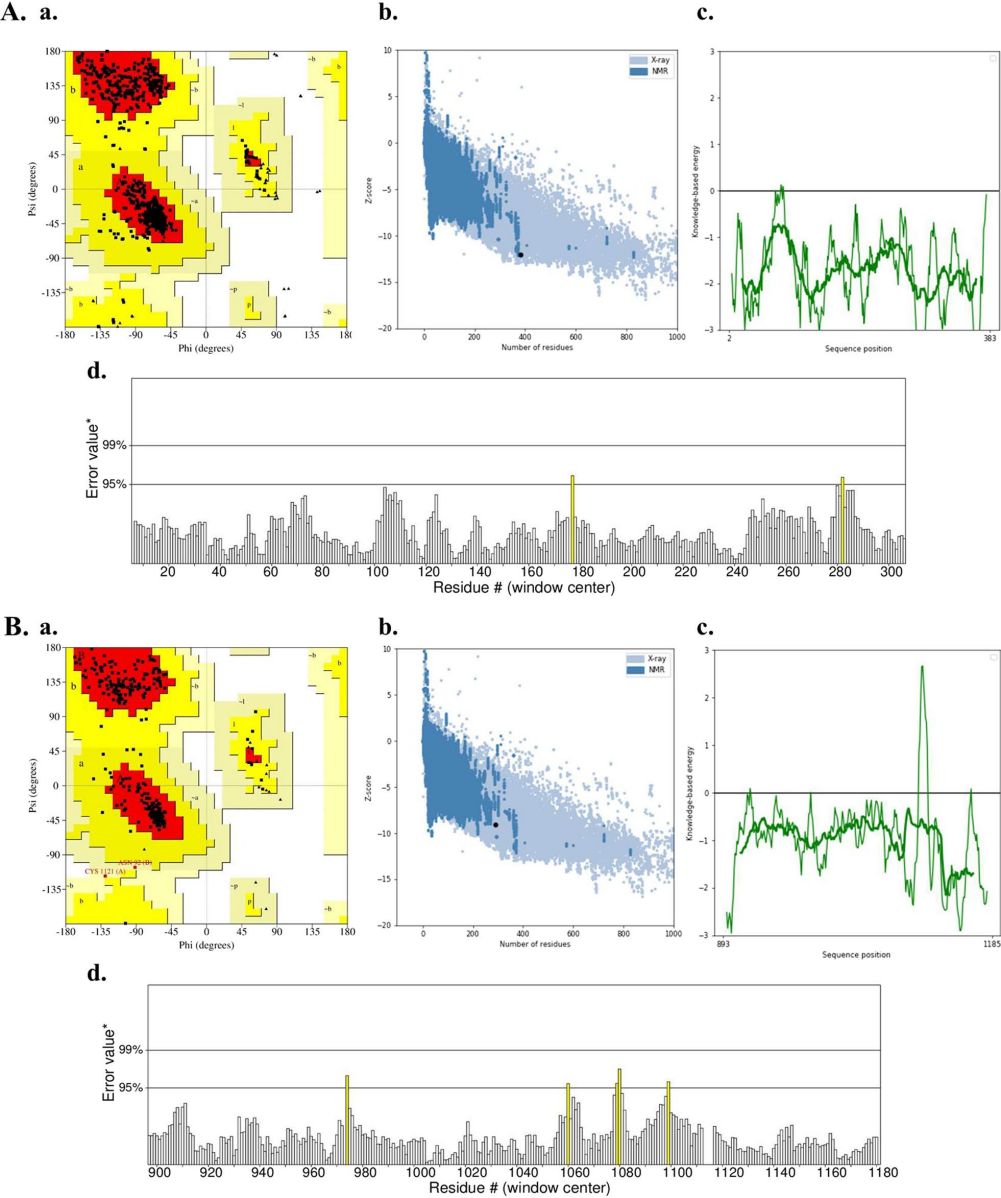
Validation of predicted structures: **A** HPV-18 E6 oncoprotein and **B** HPV-18 E7 oncoprotein; **a** Ramachandran plot, **b** Local model quality at ProSA-web, **c** Overall model quality at ProSA-web, and d. ERRAT

The accuracy of the tertiary structures of the HPV-18 E6 and E7 oncoproteins (Figs. 4A and B, respectively) was assessed and validated using the Ramachandran plot (a), ProSA-web (b and c), and ERRAT (d) servers. The evaluation results, as depicted in image a in Fig. 4A, showed that 92.8% of the amino acids in the HPV-18 E6 oncoprotein were located in the highly favored region of the Ramachandran plot, while 7.2% were in the additional allowed region, with no atypical amino acids found in the generously allowed and disallowed regions. Similarly, in the case of the HPV-18 E7 oncoprotein (image a in Fig. 4B), 92.0% of the residues were situated in the favored region, 7.3% in the additional allowed region, and 0.7% in the generously allowed region.

To further assess the quality of the structures, the ProSA-web algorithm was utilized, and the obtained Z-scores were − 12.05 for HPV-18 E6 (Image b in Fig. 4A) and − 9.09 for HPV-18 E7 (Image b in Fig. 4B). Additionally, the ERRAT server was employed to evaluate the overall quality factor of the generated models. The HPV-18 E6 oncoprotein received a quality factor of 99.6032 (images d in Fig. 4A), while the HPV-18 E7 oncoprotein had a quality factor of 96.8652 (images d in Fig. 4B). These findings indicate that the modeled structures of the HPV-18 E6 and E7 oncoproteins are reliable and can be considered as potential targets for natural photosensitizers. According to the results of SignalP—5.0, HPV-18 E6 and E7 oncoproteins do not have signal peptides cleavage sites (Sec/SPI = 0.011 and Sec/SPI = 0.0014, respectively). So, these oncoproteins are not secreted through Sec-dependent pathways. Future experiments are required to identify these secretion pathways.

### Functional classification

**Fig. 5:**
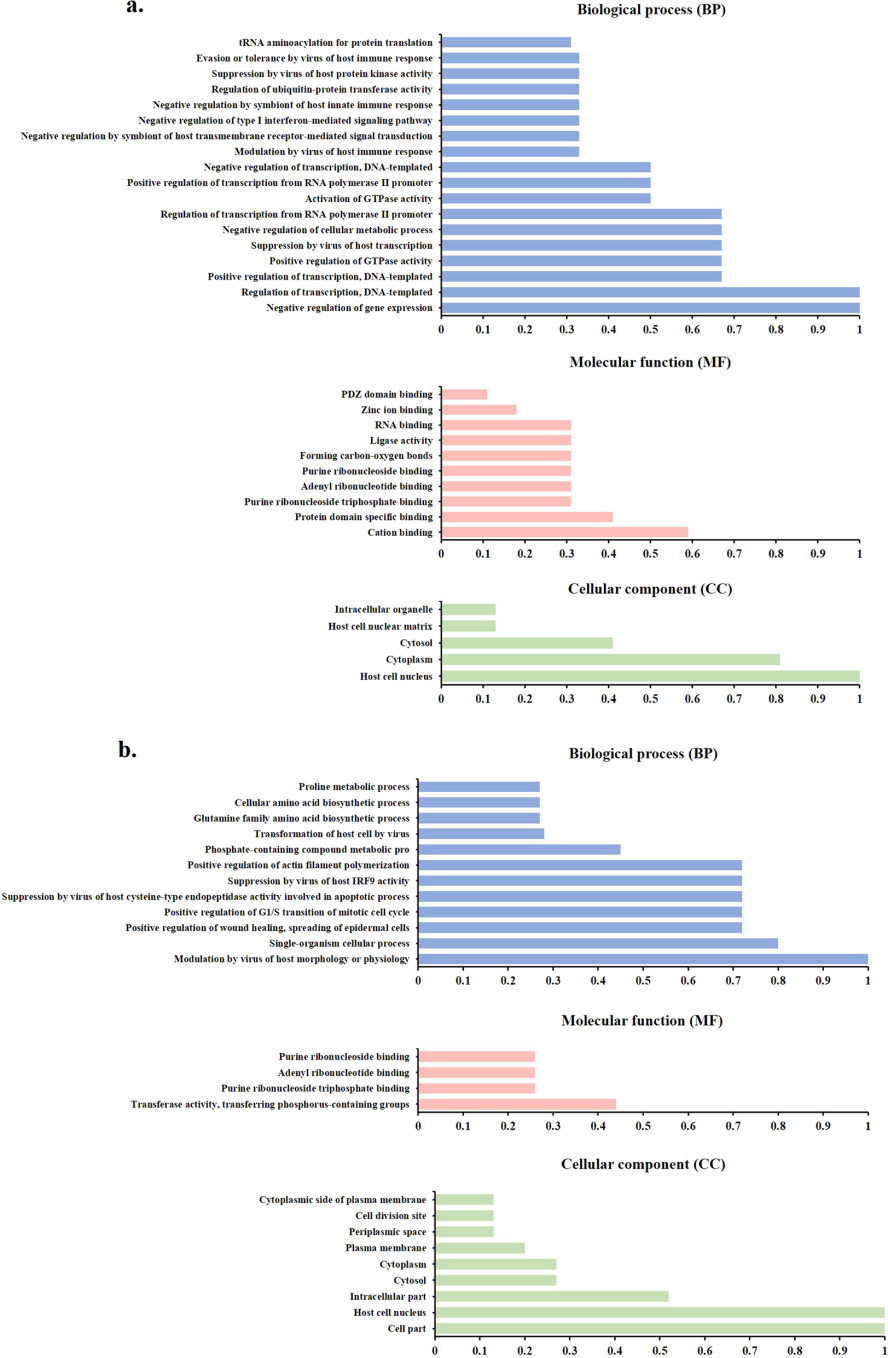
Gene Ontology (GO) enrichment analysis. The bar graphs indicate the enriched GO terms of the differentially expressed genes and the numbers of genes corresponding to each GO term. **a** HPV-18 E6 oncoprotein, **b** HPV-18 E7 oncoprotein

The refined models of the HPV-18 E6 and E7 oncoproteins were analyzed using C-I-TASSER, a structure-based method for determining the biological function of protein molecules. The analysis generated a comprehensive list of predicted Gene Ontology (GO) terms for each oncoprotein structure, which can be observed in Fig. 5. According to the analysis, the E6 oncoprotein (Fig. 5a) exhibited significant enrichment in GO terms associated with negative regulation of gene expression and regulation of transcription, DNA-templated, both with a CscoreGO value of 1.00, in the biological process (BP) category. As for the E7 oncoprotein (Fig. 5b), it demonstrated significant enrichment in a GO term related to the modulation of host morphology or physiology by the virus, with a Cscore^GO^ value of 1.00. In terms of molecular function (MF), both E6 and E7 oncoproteins displayed significant enrichment in GO terms related to cation binding and transferase activity, transferring phosphorus-containing groups, with Cscore^GO^ values of 0.59 and 0.44, respectively. Regarding cellular component (CC), the E6 oncoprotein was found to be significantly enriched in GO terms associated with the host cell nucleus, with a Cscore^GO^ value of 1.00 (Fig. 5a). On the other hand, the E7 oncoprotein exhibited significant enrichment in GO terms related to both the host cell nucleus and cell part, both with a Cscore^GO^ value of 1.00 (Fig. 5b).

### Molecular dynamics simulation

**Fig. 6:**
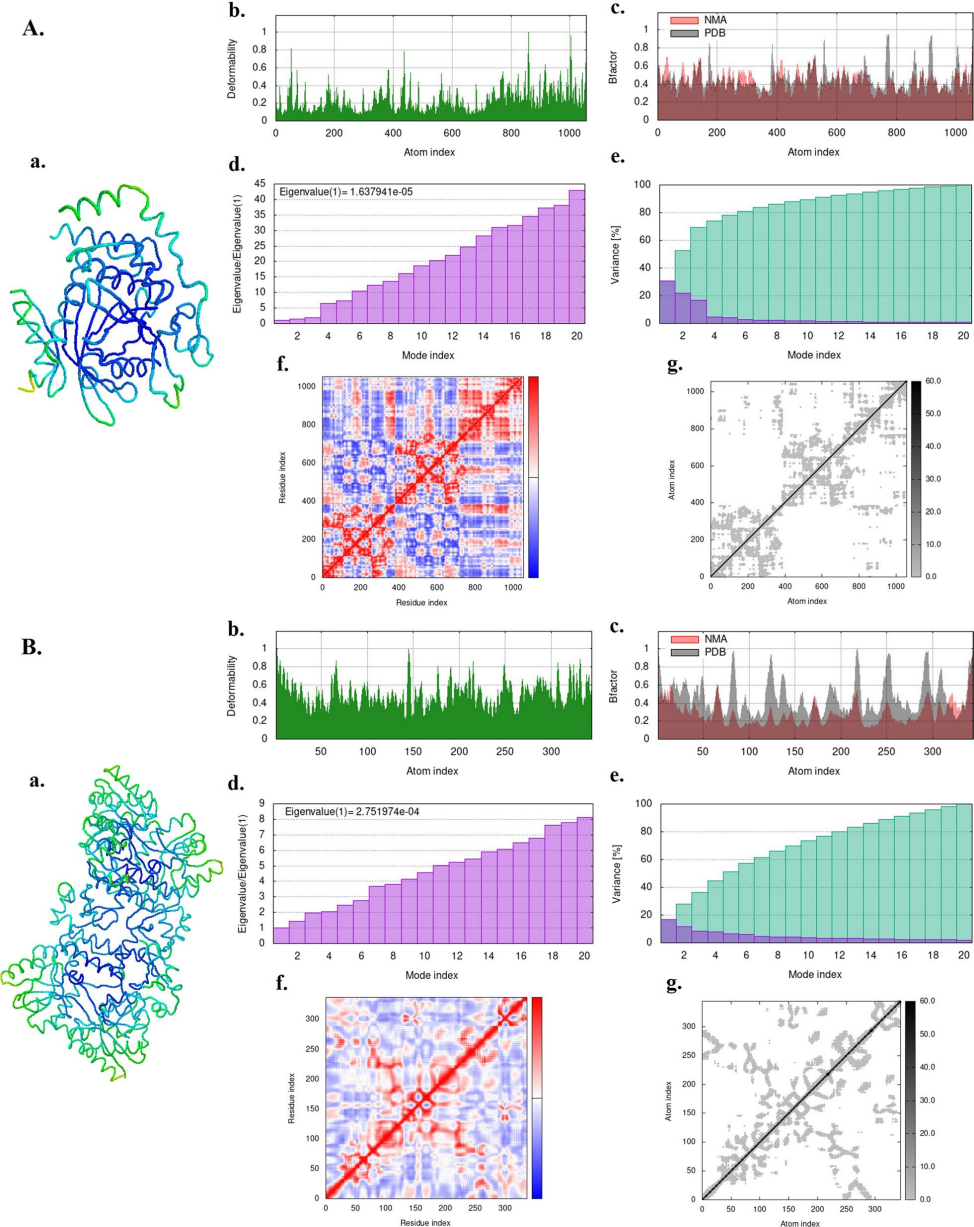
Molecular dynamic simulation: **A** HPV-18 E6 oncoprotein, **B** HPV-18 E7 oncoprotein. **a** Protein–ligand complex, **b** Deformability, **c**. B-factor values, **d** Eigenvalues, **e**. Variance (violet: individual variances, green: cumulative variances), **f** Co-variance map (residues with correlated motions in red, uncorrelated motions in white, and anti-correlated motions in blue), and **g**. Elastic network (darker grays indicate stiffer springs) of the complex

The results of the normal mode analysis (NMA) performed on the HPV-18 E6 and E7 oncoproteins are presented in Fig. 6A and B, respectively. Image a portrays the outcomes of the NMA, while image b highlights the regions in the proteins that exhibit high deformability. It is observed that the E7 protein (Image b in Figs. 6B) displays greater deformability compared to E6 (Image b in Figs. 6A), with multiple peaks indicating a deformability index of approximately 1.0. The B-factor, which indicates the flexibility of the proteins based on atomic displacement parameters, was calculated using NMA and is depicted in image c in Figs. 6A and B. Image d represents the eigenvalues of the proteins, which relate to the energy required to deform their structures. A lower eigenvalue suggests easier deformation. Specifically, the eigenvalues for HPV-18 E6 and E7 oncoproteins were 1.637941e-05 (Image d in Fig. 6A) and 2.751974e-04 (Image d in Fig. 6B), respectively. Image e showcases the variance plots, illustrating cumulative variances in green and individual variances in violet. In image f, the covariance matrix between pairs of residues is shown, where red indicates good correlation, white represents no correlation, and blue signifies anticorrelation. Furthermore, image g presents the elastic network model, which displays the connections between atom pairs and springs. Dark grey dots indicate stiffer springs, while lighter grey dots represent more flexible ones. The iMOD study conducted on the target proteins suggests that the proposed proteins are stable.

### Molecular docking

**Fig. 7:**
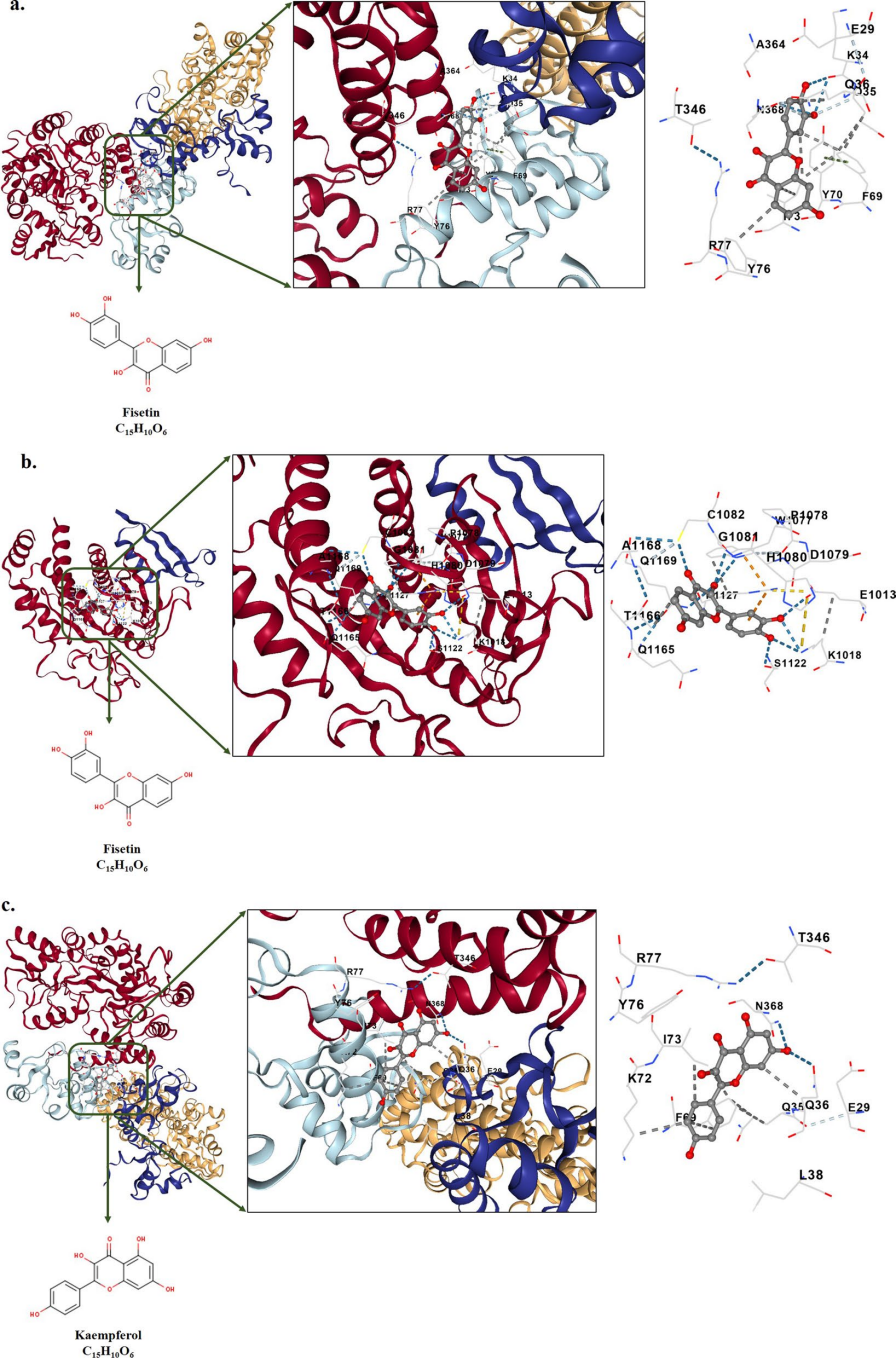

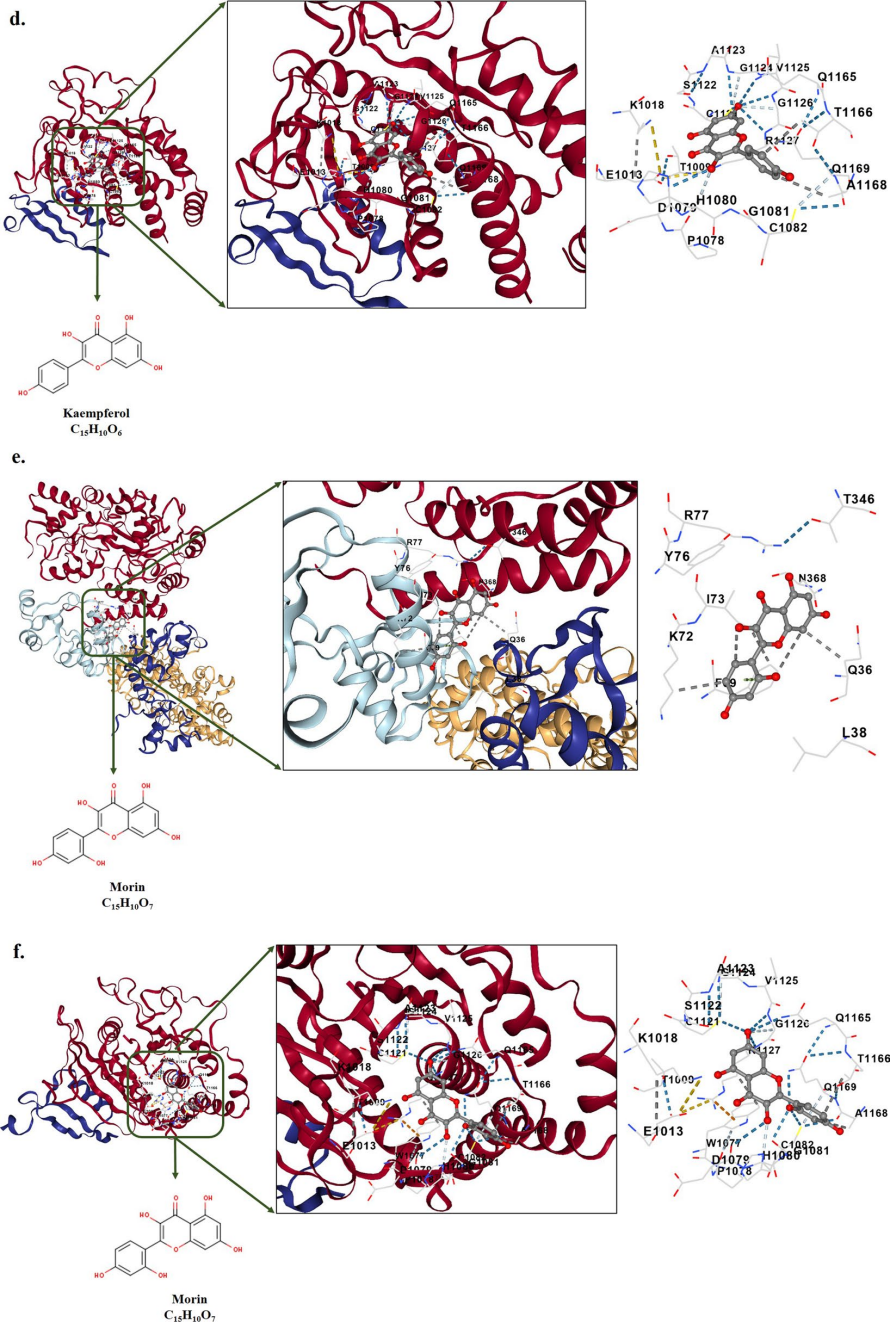

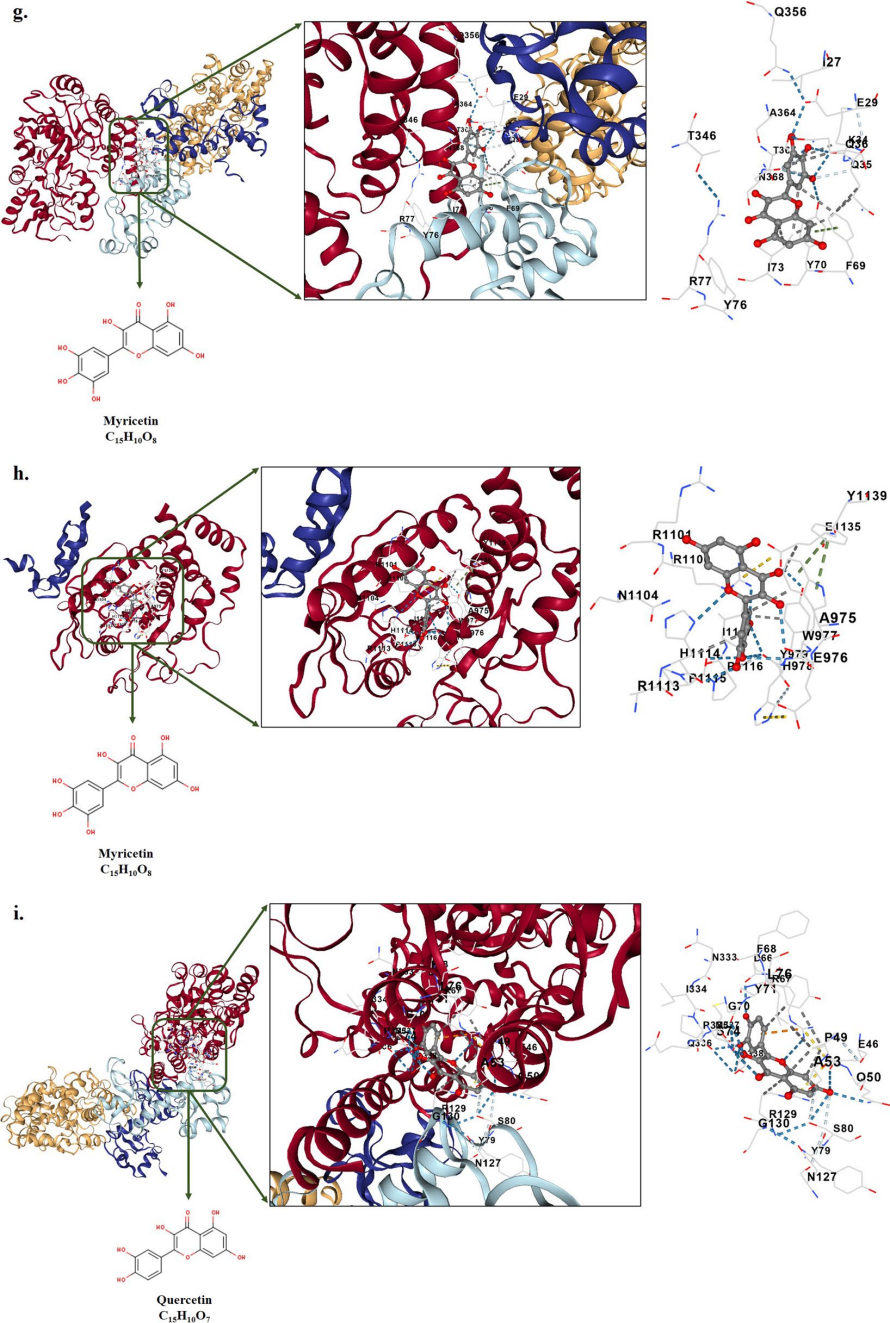

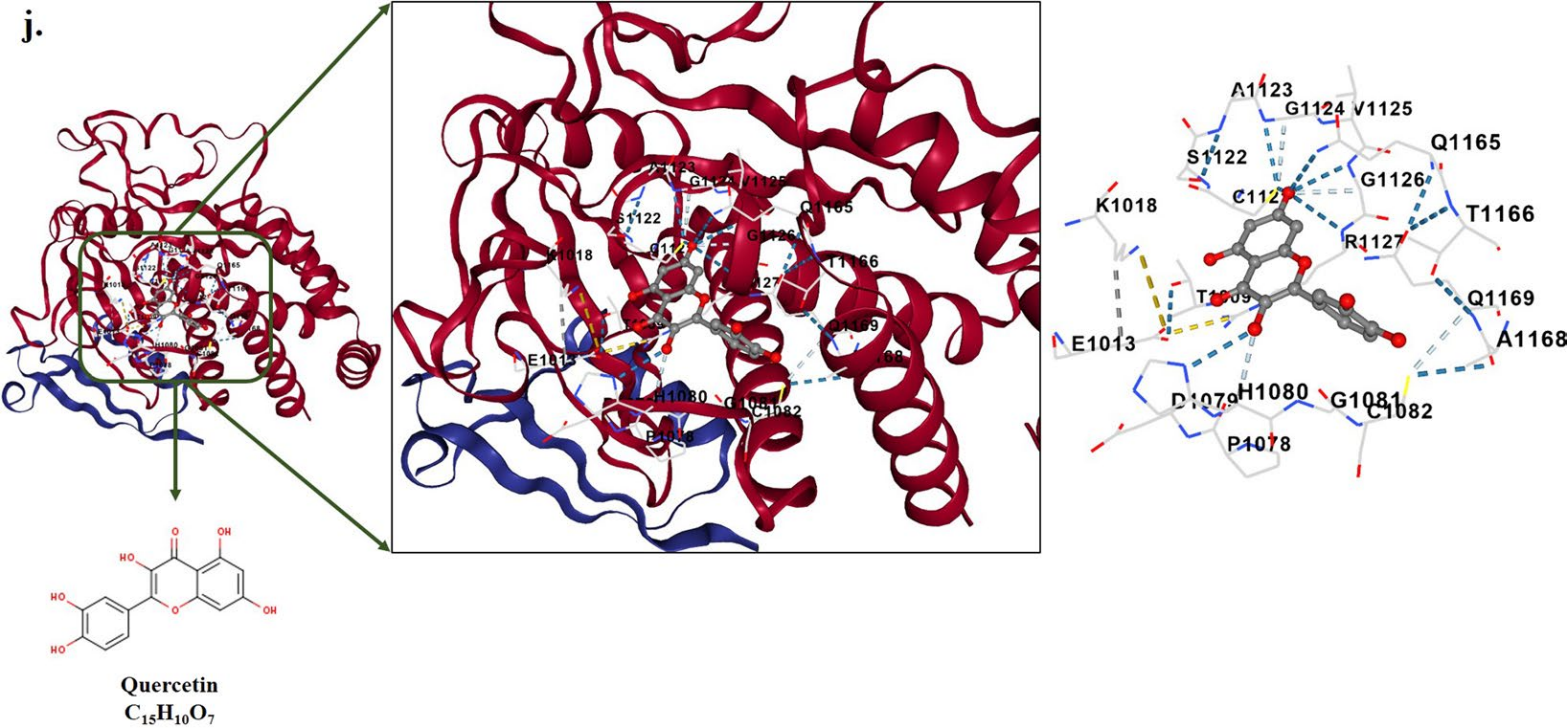
Depiction of docked ligand–protein complex along with the interaction of the amino acid residues of the protein with ligand: **a** HPV-18 E6 oncoprotein-Fisetin, **b** HPV-18 E7 oncoprotein-Fisetin, **c**. HPV-18 E6 oncoprotein-Kaempferol, **d** HPV-18 E7 oncoprotein-Kaempferol, **e**. HPV-18 E6 oncoprotein-Morin, **f** HPV-18 E7 oncoprotein-Morin, **g** HPV-18 E6 oncoprotein-Myricetin, **h** HPV-18 E7 oncoprotein-Myricetin, **i** HPV-18 E6 oncoprotein- Quercetin, and **j** HPV-18 E7 oncoprotein- Quercetin

Table 2 and Fig. 7 present a summary of the free binding energy values and various interactions of natural flavonoid glycosides with HPV-18 E6 and E7 oncoproteins. The results indicate that Kaempferol exhibited a strong binding affinity to both E6 and E7 oncoproteins, with binding energies of − 9.2 kcal/mol and − 7.9 kcal/mol, respectively. In the case of E6, Kaempferol formed interactions with several amino acid residues, including GLU45, GLU46, PRO49, GLN50, ALA53, ASP66, ARG67, GLY70, TYR71, SER74, LEU76, ILE334, PRO335, SER338, SER80, TYR81, ASN127, ARG129, and GLY130. Similarly, in the case of E7, Kaempferol interacted with ALA975, GLU976, TRP977, HIS978, TYR979, GLN1097, ARG1100, ARG1101, ARG1113, HIS1114, PRO1115, PRO1116, ILE1117, TYR1139, and HIS1143.

**Table 2:** Properties of molecular docking analysis between the HPV-18 E6 and E7 oncoproteins (target receptors) and natural flavonoid glycosides (ligands) including Fisetin, Kaempferol, Morin, Myricetin, and Quercetin

Compound Name	Molecular Formula	PubChem CID	Interactions		∆Gbinding (Kcal/mol)	
			E6	E7	E6	E7
Fisetin	C_15_H_10_O_6_	5281614	GLU45, PRO49, ARG67, PHE68, GLY70, TYR71, SER74, HIS78, ILE128, ARG129, GLY130, PRO335, GLN336, SER338, ALA339, GLU372, LEU373, GLN376, ARG383	ALA975, GLU976, TRP977, HIS978, TYR979, GLN1097, ARG1100, ARG1101, ASN1104, HIS1114, PRO1115, PRO1116, ILE1117, TYR1139, HIS1143	− 8.4	− 7.5
Kaempferol	C_15_H_10_O_6_	5280863	GLU45, GLU46, PRO49, GLN50, ALA53, ASP66, ARG67, GLY70, TYR71, SER74, LEU76, SER80, TYR81, ASN127, ARG129, GLY130, ILE334, PRO335, SER338	ALA975, GLU976, TRP977, HIS978, TYR979, GLN1097, ARG1100, ARG1101, ARG1113, HIS1114, PRO1115, PRO1116, ILE1117, TYR1139, HIS1143	− 9.2	− 7.9
Morin	C_15_H_10_O_7_	5281670	HIS65, ASP96, ALA97, ARG99, TYR100, ASN101, TYR172, GLY175, LYS176, TYR177, ILE330, MET331, PRO332, ASN333, ILE334	THR1009, GLU1013, LYS1018, TRP1077, PRO1078, ASP1079, HIS1080, GLY1081, CYS1082, CYS1121, SER1122, ALA1123, GLY1124, VAL1125, GLY1126, ARG1127, GLN1165, THR1166, ALA1168, GLN1169	− 8.4	− 7.3
Myricetin	C_15_H_10_O_8_	5281672	PRO49, ASP66, ARG67, GLY70, TYR71, LEU373, SER74, LEU76, HIS78, ARG129, GLY130, PRO335, GLN336, SER338, ALA339, GLU372, GLN376, ARG383,	ALA975, GLU976, TRP977, HIS978, TYR979, ARG1100, ARG1101, ASN1104, ARG1113, HIS1114, PRO1115, PRO1116, ILE1117, GLU1135, TYR1139	− 8.9	− 7.4
Quercetin	C_15_H_10_O_7_	5,280,459	GLU46, PRO49, GLN50, ALA53, ASP66, ARG67, PHE68, GLY70, TYR71, SER74, LEU76, TYR79, SER80, ASN127, ARG129, GLY130, ASN333, ILE334, PRO335, GLN336, MET337, SER338	ALA975, GLU976, TRP977, HIS978, TYR979, GLN1097, ARG1100, ARG1101, ASN1104, HIS1114, PRO1115, PRO1116, ILE1117, TYR1139, HIS1143,	− 8.9	− 7.5

### Evaluation of drug-likeness properties

Table 3 presents the drug-likeness properties of natural flavonoid glycosides. The results indicate that all of the natural compounds, except for Myricetin, fulfilled Lipinski's rule of five without any violations. Lipinski's rule states that for a compound to have good drug-like properties, it should have a molecular weight less than 500 g/mol, an octanol/water partition coefficient (LogP) less than 5, fewer than 5 hydrogen bond donors, fewer than 10 hydrogen bond acceptors, and a total polar surface area (TPSA) less than 140 Å^2^.

**Table 3:** Drug-likeness characteristics of natural flavonoid glycosides including Fisetin, Kaempferol, Morin, Myricetin, and Quercetin

Drug-likeness properties	Fisetin	Kaempferol	Morin	Myricetin	Quercetin
Molecular weight (g/mol)	286.24	286.24	302.24	318.24	302.24
Number of H-bond acceptor	6	6	7	8	7
Number of H-bond donor	4	4	5	6	5
Number of rotatable bonds	1	1	1	1	1
TPSA (Å^2^)	111.13	111.13	131.36	151.59	131.36
Log Po/w (iLOGP)	1.50	1.70	1.47	1.08	1.63
Lipinski’s rule of five violation	Yes; 0 violation	Yes; 0 violation	Yes; 0 violation	Yes; 1 violation	Yes; 0 violation
Bioavailability score	0.55	0.55	0.55	0.55	0.55

### Evaluation of ADMET properties

Table 4 provides information on the ADMET (absorption, distribution, metabolism, excretion, and toxicity) properties of natural flavonoid glycosides. The absorption level of these compounds was determined using parameters such as intestinal absorption (human), skin permeability, Caco-2 permeability, and their interactions with P-glycoprotein. A Papp coefficient greater than 8 × 10^–6^ indicates high Caco-2 permeability and easy absorption. Notably, none of the tested compounds showed poor Caco-2 permeability, and all were predicted to have high absorption levels for intestinal absorption (human). Skin permeability, assessed by the log Kp value, indicated that none of the compounds had low skin permeability.

**Table 4:** Predicted ADMET properties of natural flavonoid glycosides including Fisetin, Kaempferol, Morin, Myricetin, and Quercetin

Model Name	Fisetin	Kaempferol	Morin	Myricetin	Quercetin
Absorption					
Water solubility (log mol/L)	− 3.181	− 3.04	− 2.978	− 2.915	− 2.925
Caco2 permeability (log Papp in 10^–6^ cm/s)	0.058	0.032	− 0.294	0.095	− 0.229
Intestinal absorption (human) (% Absorbed)	83.752	74.29	75.408	65.93	77.207
Skin permeability (log Kp)	− 2.735	− 2.735	− 2.735	− 2.735	− 2.735
P-glycoprotein substrate	Yes	Yes	Yes	Yes	Yes
P-glycoprotein I inhibitor	No	No	No	No	No
P-glycoprotein II inhibitor	No	No	No	No	No
Distribution					
VDss (human) (log L/kg)	0.718	1.274	1.229	1.317	1.559
Fraction unbound (human) (Fu)	0.166	0.178	0.214	0.238	0.206
BBB permeability (log BB)	− 1.039	− 0.939	− 1.18	− 1.493	− 1.098
CNS permeability (log PS)	− 2.282	− 2.228	− 3.389	− 3.709	− 3.065
Metabolism					
CYP2D6 substrate	No	No	No	No	No
CYP3A4 substrate	No	No	No	No	No
CYP1A2 inhibitor	Yes	Yes	Yes	Yes	Yes
CYP2C19 inhibitor	No	No	No	No	No
CYP2C9 inhibitor	Yes	No	No	No	No
CYP2D6 inhibitor	No	No	No	No	No
CYP3A4 inhibitor	No	No	No	No	No
Excretion					
Total clearance (log ml/min/kg)	0.421	0.477	0.486	0.422	0.407
Renal OCT2 substrate	No	No	No	No	No
Toxicity					
AMES toxicity	No	No	No	No	No
hERG I inhibitor	No	No	No	No	No
hERG II inhibitor	No	No	No	No	No
Hepatotoxicity	No	No	No	No	No
Skin sensitization	No	No	No	No	No

^*^ Papp: apparent permeability coefficient, AMES: assay of the ability of a chemical compound to induce mutations in DNA, Kp: skin permeability, VDss: distribution volume, Fu: fraction unbound, BBB: blood–brain barrier, BB: blood–brain, CNS: central nervous system, PS: permeability-surface area, OCT2: Organic cation transporter 2, hERG: human ether-a-go-go-related gene

In terms of P-glycoprotein interactions, Fisetin, Kaempferol, Morin, Myricetin, and Quercetin were identified as substrates, suggesting that they may be actively excreted by P-glycoprotein. However, none of the compounds were predicted to be P-glycoprotein inhibitors.

The distribution of compounds was evaluated using parameters such as distribution volume (VDss), blood–brain barrier (BBB) permeability (log BB), fraction unbound (human), and CNS permeability. A lower VDss value indicates relatively low distribution volume, while a higher VDss value suggests relatively high distribution volume. The results revealed that all natural flavonoid glycosides had high distribution volumes.

For BBB permeability, a log BB greater than 0.3 indicates easy crossing of the BBB, while a log BB less than − 1 suggests difficulty. Among the compounds, Fisetin (− 1.039), Kaempferol (− 0.939), Morin (− 1.18), Myricetin (− 1.493), and Quercetin (− 1.098) were predicted to have difficulty crossing the BBB. Regarding CNS permeability, Morin (− 3.389), Myricetin (− 3.709), and Quercetin (− 3.065) were predicted to be unable to penetrate the CNS, while Fisetin (− 2.282) and Kaempferol (− 2.228) were predicted to have the ability to do so.

The compounds were also evaluated for their interactions with cytochrome P450 enzymes, specifically CYP2D6, CYP3A4, CYP1A2, CYP2C9, and CYP2C19. None of the compounds were substrates for CYP2D6 and CYP3A4, but all were predicted to be inhibitors of CYP1A2. Furthermore, it was predicted that all the compounds would function as inhibitors for CYP2D6, CYP3A4, and CYP2C19. However, out of these naturally occurring flavonoid glycosides, only Fisetin displayed inhibitory effects on CYP2C9 (Table 4).

The clearance of compounds was assessed based on their molecular weight and hydrophilicity, with Morin having the highest total clearance, followed by Kaempferol, Myricetin, Fisetin, and Quercetin. None of the compounds showed toxicity in the AMES test or hepatotoxicity, according to the prediction results. Additionally, they were not found to inhibit the hERG channel, suggesting a lack of cardiotoxicity, nor did they show any skin sensitization. In summary, the predicted results suggest that all of the tested compounds exhibit similar ADMET characteristics.

## Discussion

Oropharyngeal cancer associated with HPV infection is a significant concern (Lechner et al. 2022). aPDT is a treatment method that has both antiviral and anticancer properties. It is a safe and easily applicable method that may have a broad range of photoantimicrobial activity, unlike traditional agents. The antiviral/anticancer mechanism of aPDT involves the generation of ROS that can damage the target cell membrane, proteins, and DNA, leading to cell death. In addition to its direct antimicrobial/anticancer effects, aPDT can also stimulate the immune system to enhance the host's natural defense mechanisms against microbial infections (Fekrazad et al. 2021; Kolarikova et al. 2023).

The photosensitizer plays a crucial role in aPDT by absorbing light energy and initiating the production of ROS, which ultimately leads to the destruction of targeted cells. Selecting the right photosensitizer is essential for the success of aPDT in the treatment of different ailments, such as cancer and viral infections. (Fekrazad et al. 2021). Different types of photosensitizers are available, including synthetic and natural compounds, as well as metal-based molecules. Natural photosensitizers are a promising alternative to synthetic and metal-based photosensitizers because they are usually less toxic and more biocompatible (Polat and Kang 2021). Using natural photosensitizers in aPDT offers several advantages. Firstly, the natural photosensitizers are often less expensive and more readily available than synthetic ones. Secondly, natural photosensitizers can be derived from diverse sources, including plants, algae, and bacteria, which can provide a diverse range of photosensitizers with different properties. Thirdly, natural photosensitizers are usually biodegradable and have a lower environmental impact than synthetic ones (Villacorta et al. 2017; Siewert and Stuppner 2019; Polat and Kang 2021). Furthermore, natural photosensitizers have shown promise in enhancing the efficiency of aPDT.

Flavonoids have been extensively investigated for their ability to function as photosensitizers in various domains. Their distinctive chemical structure, featuring a chromophore comprising conjugated double bonds and phenolic groups, enables them to absorb light within the visible range of the electromagnetic spectrum and undergo photoexcitation. A few studies have demonstrated the photophysical properties of flavonoids and their ability to act as photosensitizers (Kulbacka et al. 2016; Motallebi et al. 2020; Sabagh et al. 2020; Zdyb and Krawczyk 2019). A study by Motallebi et al. (2020) investigated the effect of rutin as flavonoid compound on photodynamic inactivation against *Pseudomonas aeruginosa* and *Staphylococcus aureus*. Their findings revealed that the combination of rutin with methylene blue and aPDT resulted in a greater decrease in the number of bacteria in both planktonic condition and bacterial biofilm production compared to the use of methylene blue alone. Pourhajibagher and Bahador (2023a) employed computational methods to explore the potential of Quercetin as a natural flavonoid glycoside in targeted aPDT against the D8L protein of the Monkeypox virus. The findings indicated a strong affinity of this photosensitizer to the D8L protein, suggesting its possible use as supplementary treatments for Monkeypox disease. In another study, they showed that aPDT using Quercetin reduced microbial biofilm growth and the expression of the bap gene involved in *Acinetobacter baumannii* biofilm formation (Pourhajibagher et al. 2022).

In the current study, natural flavonoid glycosides were utilized as photosensitizers to inhibit the oropharyngeal HPV-18 E6 and E7 oncoproteins. Fisetin, a flavonoid that naturally occurs in various fruits and vegetables, possesses diverse properties similar to other flavonoids, including antioxidant, antiviral, antibacterial, and anti-inflammatory activities (Ravula et al. 2021; Park et al. 2022). Moreover, Fisetin has demonstrated anticancer effects in both in vitro and in vivo studies. Treatment with Fisetin has been shown to impede the growth of lung cancer cells in humans (Khan et al. 2012). Additionally, Fisetin has been found to suppress the proliferation and migration of human oral squamous cell carcinoma, leading to inhibition of tumor growth (Ravula et al. 2021). Kaempferol, another flavonoid present in various plants, has garnered attention for its potential health benefits including anticancer, anti-inflammatory, antioxidant, antibacterial, and antiviral properties (Devi et al. 2015; Lei et al. 2019; Li et al. 2021; Jiang et al. 2022). Morin, a flavonol pigment derived from different plants, particularly the Moraceae family, exhibits a wide range of pharmacological properties, including anticancer effects (Nandhakumar et al. 2012; Caselli et al. 2016; Rajput et al. 2021). Myricetin, a significant polyphenolic flavonoid found in numerous plants, possesses the ability to inhibit the growth of both bacteria and viruses. (Chen et al. 2019). Studies have revealed that Myricetin can impede pseudorabies virus infection by directly deactivating the virus and stimulating the host's antiviral defense (Hu et al. 2022). Previous studies have indicated that the main mechanisms of action for Myricetin involve direct viral killing and inhibition of viral adsorption or penetration (Li et al. 2020; Wang et al. 2023). This makes it a promising candidate for preventing virus infections or for use in combination with other drugs targeting viral replication stages (Hu et al. 2022). Additionally, Quercetin, a flavonoid commonly found in fruits and vegetables, is well-known for its antioxidant, antiviral, antimicrobial, and anti-inflammatory properties (Di Petrillo et al. 2022). Numerous investigations have suggested that Quercetin has the potential to serve as an antiviral agent by preventing initial stages of virus infection, interacting with proteases involved in viral replication, and reducing inflammation resulting from infections (Gansukh et al. 2016; Di Petrillo et al. 2022).

According to the literature, no research has been conducted to investigate the potential of using natural flavonoid glycosides in aPDT. Thus, in this *in silico* study, we aim to assess the antiviral impact of aPDT mediated by natural flavonoid glycosides as inhibitors against oropharyngeal HPV-18 E6 and E7 oncoproteins using various bioinformatics methods. HPV-18 E6 and E7 oncoproteins play a crucial role in driving HPV-associated oropharyngeal cancer, making them attractive targets for the development of novel therapeutic strategies. In this study, various computational analysis methods and modeling simulations were employed, and the results indicated the predicted stability of these proteins. The GO annotation revealed that E6 was associated with 33 functional terms, including 18 terms for biological processes, 10 terms for molecular functions, and 5 terms for cellular components. The biological process group primarily consisted of genes involved in the negative regulation of gene expression and the regulation of transcription, DNA-templated. The molecular function terms were associated with cation binding and protein domain specific binding. The majority of the cellular component genes were found in the host cell nucleus and cytoplasm. The E7 genes were involved in 9 biological processes, primarily modulation of host morphology/physiology and single-organism cellular processes. The 4 molecular functions were associated with transferase activity transferring phosphorus-containing groups. The 9 cellular components were mainly located in the cell parts and host cell nucleus. Overall, the GO analysis found that E6 genes were involved in gene regulation while E7 genes played roles in altering host cell morphology and physiology. The two groups of genes had distinct molecular functions and cellular localizations. Moreover, the *in silico* molecular docking study suggests that natural flavonoid glycosides, particularly Kaempferol, have potential as photosensitizing agents against HPV-18 in the oropharynx. The docking analysis showed that these natural compounds had a high probability of binding to the active sites of the E6 and E7 oncoproteins of HPV-18.

Herein, the drug-likeness and bioavailability of the compounds under investigation were assessed using ADMET profiling. The utilization of *in silico* ADMET profiling serves as a valuable tool in predicting the pharmacological and toxicological properties of potential drug candidates, particularly during the pre-clinical stages. *In silico* models have been developed to improve these predictions, which can enhance drug optimization and prevent costly late-stage failures. In the case of oral photosensitizers, rapid and thorough absorption from the gastrointestinal tract, along with safe elimination from the body without causing harm, is of utmost importance. The appropriateness of a photosensitizer's ADMET profile determines its therapeutic applications, and simulation can help identify safer inhibitors with minimal side effects. Efficiency and a satisfactory ADMET profile are the primary criteria for a photosensitizer, emphasizing the importance of calculating pharmacokinetic attributes during hit discovery and identification. The process of investigating the effectiveness of photosensitizer as an antimicrobial compound involves multiple stages, beginning with identifying the target and concluding with predicting how it will behave in the body (ADMET prediction). To gain a deeper understanding of how natural flavonoid glycosides pass through the body, the ADMET parameters of pharmacokinetics (Absorption, Distribution, Metabolism, Excretion, and Toxicity) were measured. The results revealed that all compounds exhibited high Caco-2 permeability, indicating easy absorption, with no compounds showing poor Caco-2 permeability. Among the compounds, Fisetin, Kaempferol, Morin, Myricetin, and Quercetin were identified as substrates of P-glycoprotein, but none were predicted to inhibit P-glycoprotein. Furthermore, the distribution volumes of Fisetin, Kaempferol, Morin, Myricetin, and Quercetin were determined to be high. The interaction of the compounds with cytochrome P450 enzymes was also assessed. While none of the compounds were substrates for CYP2D6 and CYP3A4, they were predicted to inhibit CYP1A2, CYP2D6, CYP3A4, and CYP2C19, with only Fisetin displaying inhibitory effects on CYP2C9. The molecular weight and hydrophilicity of the compounds influenced drug elimination, with Morin having the highest total clearance. The tested compounds demonstrated negative results in terms of toxicity in the AMES test, hepatotoxicity, cardiotoxicity, and skin sensitization.

The findings reported herein demonstrate the versatility of flavonoid glycosides for selective binding to active sites of HPV-18 E6 and E7 oncoproteins. To demonstrate the effectiveness of flavonoid glycosides as photosensitizers in targeted PDT against HPV, in vitro experimental assays are required. The use of animal models is also necessary to optimize the conditions for HPV inactivation specifically. Targeted PDT is indeed an emerging strategy in the field of PDT, showing great potential for local infectious diseases and malignant tumors. In targeted PDT, photosensitizers can selectively bind to specific targets of infectious agents and cancerous cells, and upon light activation, produce ROS that leads to microbial cell inactivation and apoptosis of cancer cells. The selectivity of photosensitizers for infectious agents and cancer cells over host cells, accurate delivery of the photosensitizers into the infected and cancer cells area, and PDT dose adjustment help minimize side effects and give PDT an advantage over conventional treatments. However, there are still only a few reports about the use of targeted PDT. In a recent study Pourhajibagher and Bahador (2023b) revealed that resveratrol, emodin, and pterin could efficiently interact with the M^Pro^ of Severe Acute Respiratory Syndrome-Coronavirus-2 (SARS-CoV-2) and these natural photosensitizers may be considered a potential SARS-CoV-2 M^Pro^ inhibitor to control COVID-19 following activation by light of a specific wavelength. In the other study (Thomas-Moore et al. 2023), galactose-polyethylene glycol-3-/chlorin e6-polyethylene glycol-4-gold nanoparticles (Gal-PEG3-/ce6-PEG4-AuNPs) showed selective interactions with breast cancer cell lines, inducing targeted cell death through PDT following activation with a 633 nm laser. In the cancer cell lines galectins (lectins that bind β-d-galactosides) were identified as the specific cellular targets of Gal-PEG3-/ce6-PEG4-AuNPs. No significant interaction was detected with the non-cancer cell line MCF-10A, demonstrating selective cell killing of the cancer cell line with Gal-PEG3-/ce6-PEG4-AuNPs. The selective cell killing of breast cancer cells demonstrates the potential of using this approach for targeted PDT. Therefore, targeted PDT with the design of photosensitizers against specific cellular structures or receptors has great potential as a novel strategy for precisely targeting microbial cells and tumor cells, reducing side effects on normal tissues. The upcoming investigations will entail assessing the impact of targeted PDT in animal models to aid in refining the conditions for specifically targeting microbial and cancer cell destruction.

Collectively, these predicted findings indicate that all the compounds under investigation share similar ADMET characteristics. Thus, the computational modeling indicates that flavonoid glycosides like Kaempferol may be promising photosensitizer candidates for treating oropharyngeal HPV-18 infections due to their predicted affinity for the virus's transforming proteins. In summary, this study supports further exploration of these naturally occurring compounds as novel targeted phototherapy agents against this type of HPV.

Although aPDT is a promising approach for the treatment of local infections, there are limitations associated with the experimental validation of aPDT against oropharyngeal HPV, as well as potential future directions for further research. The major limitation is the lack of optimized protocols for aPDT against HPV. Parameters such as the type and concentration of the photosensitizer, light source characteristics, treatment duration, and timing of light activation need to be optimized and standardized to ensure consistent and reproducible results. Future research should focus on developing guidelines and protocols for aPDT that can be easily implemented in clinical settings. Additionally, HPV has multiple types, which may exhibit variations in their susceptibility to aPDT. Experimental validation should take into evaluation the effectiveness of aPDT against common types of oropharyngeal HPV individually and in combination. Additionally, the evaluation of combinatory strategies involving aPDT is essential to assess its effectiveness in combating oropharyngeal HPV infections. Furthermore, ongoing research is focusing on the identification of specific photosensitizers and light parameters that can optimize the antimicrobial effects of aPDT against HPV. These efforts aim to address the current limitations and advance the potential use of aPDT as an adjunctive treatment for oropharyngeal HPV infections.

## Data Availability

All data produced or analyzed during this study are included in this article.
